# Polar Metallicity Controlled by Epitaxial Strain Engineering

**DOI:** 10.1002/advs.202408329

**Published:** 2024-08-29

**Authors:** Mingdong Dong, Yichi Zhang, Jing‐ming Cao, Haowen Chen, Qiyang Lu, Hong‐fei Wang, Jie Wu

**Affiliations:** ^1^ Department of Physics School of Science Westlake University Hangzhou 310030 China; ^2^ Research Center for Industries of the Future Westlake University Hangzhou 310030 China; ^3^ Key Laboratory for Quantum Materials of Zhejiang Province School of Science Westlake University Hangzhou 310030 China; ^4^ School of Physics Zhejiang University Hangzhou 310027 China; ^5^ Department of Chemistry School of Science Westlake University Hangzhou 310030 China; ^6^ School of Engineering Westlake University Hangzhou 310030 China

**Keywords:** epitaxial strain engineering, nickelate, polar metal, second harmonic generation, thickness wedge

## Abstract

The discovery of polar metal opens the door to incorporating electric polarization into electronics with the potential to invigorate next‐generation multifunctional electronic devices. Especially, electric polarization can be induced by geometric design in non‐polar perovskite oxides. Here, the epitaxial strain exerted on the deposited single‐crystalline NdNiO_3_ thin films is systematically varied in both sign and amplitude by choosing substrates with different lattice mismatch. The pseudocubic NdNiO_3_(111) film, which is non‐polar in its bulk state, is induced to be polar under both compressive and tensile strain. The fine‐tuning of epitaxial strain is realized by continuously varying the film thickness using the “thickness‐wedge” growth technique, and from the elucidated thickness dependence, the electric polarization and metallicity can be further optimized. Moreover, transitioning from isotropic to anisotropic epitaxial strain gives rise to an ideal polar metal state in the pseudocubic NdNiO_3_(102) film on an orthorhombic substrate, achieving a remarkably low resistivity of 173 µΩ cm at room temperature. The metal–insulator transition in NdNiO_3_ is completely suppressed and the polar metal state becomes the ground state at all temperatures. These results demonstrate alluring possibilities of induction and manipulation of both electric polarization and electric transport properties in functional perovskite oxides by epitaxial strain engineering.

## Graphical abstract

An ideal polar metal state is achieved in the pseudocubic NdNiO_3_(102) film under an anisotropic compressive epitaxial strain. The effect of strain on polar metallicity is systematically studied by varying both the sign and amplitude of the strain. This paves the way to induction and manipulation of electric polarization in functional perovskite oxides by epitaxial strain engineering.

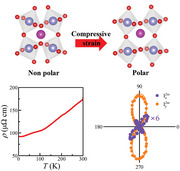

## 1 Introduction

Most ferroelectric materials are insulators that greatly limit their applications in electronics.^[^ ^1^, ^2^, ^3^ ^]^ It is a primary goal to find electric polarized materials that can conduct current as well so that sophisticated electronics‐based functions can be realized by manipulating the polarization.^[^ ^4^ ^]^ Although electric polarization and metallicity used to be considered mutually exclusive, recent discoveries of polar metals have overturned this belief and stimulated vigorous interests in the field.^[^ ^4^ ^]^ Till now, coexistence of polarization and metallicity has been identified in perovskite oxides (e.g., BaTiO_3−_ *_δ_*,^[^ ^5^ ^]^ LiOsO_3_^[^ ^6^ ^]^), layered perovskites (e.g., Ca_3_Ru_2_O_7_^[^ ^7^ ^]^), 2D materials (e.g., WTe_2_^[^ ^8^ ^]^), and other compounds (e.g., LiGaGe^[^ ^9^ ^]^).

Among approaches to realize polar metals, the induction of polarization by geometric design is a promising route.^[^ ^10^, ^11^, ^12^, ^13^, ^14^ ^]^ It has been demonstrated that the perovskite oxide NdNiO_3_, which is non‐polar in its bulk state,^[^ ^15^ ^]^ can be converted to a polar metal when it is deposited onto LaAlO_3_(111) surface.^[^ ^11^ ^]^ The titling and rotating of the corner‐connected NiO_6_ octahedra in NdNiO_3_ are constrained by epitaxial strain at the interface that results in the breaking of the inversion symmetry by forcing Nd cations to be displaced from the lattice center.^[^ ^11^, ^12^ ^]^ This finding paves the road to a variety of exciting possibilities, including induction of polarization in functional perovskite oxides,^[^ ^16^, ^17^ ^]^ for example, ferromagnetic or antiferromagnetic manganates and high‐temperature superconducting cuprates. Toward these goals, it is vital to scrutinize this effect under different types and amplitudes of epitaxial strain and optimize polar metallicity by fine tuning the interfacial strain. In particular, so far, the induced polar metallicity in NdNiO_3_ is impaired at temperatures lower than 150 K by the metal‐insulator transition (MIT) that persists in the presence of isotropic in‐plane strain.^[^ ^11^ ^]^ Whether MIT can be suppressed to consistently maintain a polar metal ground state at all temperatures is an intriguing and unresolved challenge.

## 2 Results

Taking NdNiO_3_ as the model system, we exploit advanced film synthesis skills to engineer the epitaxial strain in single crystalline NdNiO_3_ films by a combination of two methods. One is to vary the lattice mismatch between NdNiO_3_ films and the selected substrates to impose different types and amplitudes of epitaxial strain. The other is to implement the “wedge‐growth” technique to continuously vary film thickness in a desired range on a single substrate. As the epitaxial strain comes from the interface, its effect decays with film thickness. We effectively modulate the strain in NdNiO_3_ films by combining these two methods and elaborate its effect on electric polarization and metallicity by the second harmonic generation (SHG) polarimetry and electric transport measurements.

### 2.1 Film Synthesis and Strain Characterization

**Figure 1:**
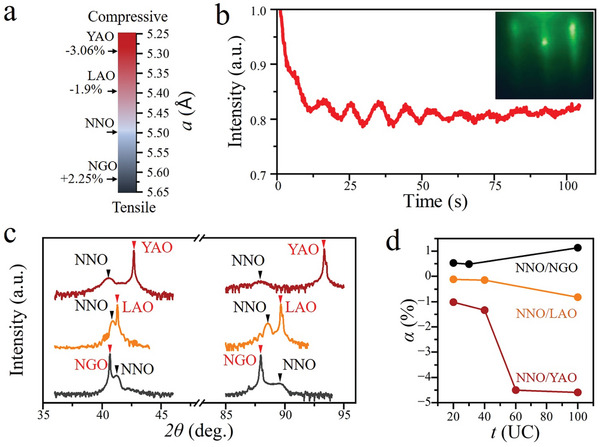
Epitaxial growth and strain modulation of pesudocubic NdNiO_3_(111) (NNO) films on LaAlO_3_(111) (LAO), YAlO_3_(101) (YAO), and NdGaO_3_(101) (NGO) substrates. a) The in‐plane lattice constant *a* along the pesudocubic $\left[1\bar{1}0\right]$ direction for YAlO_3_, LaAlO_3_, NdNiO_3_, and NdGaO_3_ bulk crystals. The corresponding lattice mismatch *α* for three substrates compared to NdNiO_3_ is marked on the left. α ≡ (*a*_substrate_ − *a*_NNO_)/*a*_NNO_. b) The oscillations in RHEED spot intensity as a function of the deposition time during deposition of NdNiO_3_ onto LaAlO_3_(111). The inset is the RHEED pattern taken on 10 UC NdNiO_3_ film. c) XRD *2θ−ω* scans of NdNiO_3_ films grown on LaAlO_3_(111), YAlO_3_(101), and NdGaO_3_(101) substrates. The black and red arrows denote the peaks corresponding to the NdNiO_3_ film and the substrate, respectively. d) The dependence of *α* on the film thickness *t*. *α* for 100 UC NdNiO_3_ on three substrates approximately reaches the bulk values, indicating the epitaxial strain is relaxed for 100 UC NdNiO_3_ films.

To modulate the titling and rotation of the Ni─O octahedral, the strain exerted on the NdNiO_3_ lattice must satisfy certain requirements of symmetry. Density functional theory calculation showed that the strain along the pseudo‐cubic NdNiO_3_(111) plane is effective in twisting the lattice and inducing electric polarization.^[^ ^11^ ^]^ We searched the crystallographic database and selected LaAlO_3_(111), YAlO_3_(101) and NdGaO_3_(101) as the epitaxial substrates. The modest lattice mismatch between NdNiO_3_(111) and the substrates’ surfaces suggested nice epitaxial growth might be achieved (**Figure** **1**a). Meanwhile, the epitaxial strain was compressive on LaAlO_3_(111) and YAlO_3_(101) substrates but tensile on NdGaO_3_(101) substrate,^[^ ^18^, ^19^, ^20^ ^]^ enabling us to systematically study the effects of the strain.

Single crystalline pseudo‐cubic NdNiO_3_(111) films were grown on top of all these three substrates by pulsed laser deposition (PLD) technique. The film quality was monitored in real time during growth by in‐situ reflection high energy electron diffraction (RHEED), and the nice layer‐by‐layer growth mode was verified by the periodic oscillations in the intensity of RHEED spots (Figure 1b). The crystal quality of NdNiO_3_ films was also characterized by ex‐situ X‐ray diffraction (XRD). The X‐ray reflectivity and XRD rocking curve confirm that NdNiO_3_ films on different substrates were all single crystalline with good quality. Concomitantly, atomic force microscopy showed the morphology of NdNiO_3_ films was quite flat with root mean square less than one atomic step. These results are included in the Supporting Information.

When the NdNiO_3_ film is thin enough, the epitaxial strain is not relaxed. Then, the in‐plane lattice of thin films follows that of the substrate so the out‐of‐plane lattice constant *c* of NdNiO_3_ varies accordingly. XRD spectrums (Figure 1c) show that compared to the bulk NdNiO_3_, *c* of NdNiO_3_ films on LaAlO_3_(111) and YAlO_3_(101) increase as its in‐plane lattice shrink in response to the compressive strain imposed by LaAlO_3_(111) and YAlO_3_(101) substrates; while *c* of NdNiO_3_/NdGaO_3_(101) decreases as the in‐plane strain becomes tensile on NGO substrate. Combining results of RHEED and XRD, we conclude that single crystalline pseudocubic NdNiO_3_(111) films are epitaxially grown on LaAlO_3_(111), YAlO_3_(101), and NdGaO_3_(101) substrates.

As the film gets thicker, its lattice eventually should relax to its bulk state. Thus, the effect of epitaxial strain is expected to diminish with film thickness. We do the reciprocal space mapping (RSM) of both NdNiO_3_ films and substrates to measure their in‐plane lattice constants. We find that even for thick films, for example, 100 unit cell (UC), there is no sign of phase separation along the *c*‐axis and the whole film has only one set of reciprocal lattice. Concomitantly, the in‐plane lattice mismatch *α* between the NdNiO_3_ films and substrates is measured as a function of film thickness (Figure 1d). For all three types of substrates, *α* is relatively small for thin NdNiO_3_ films (*t* = 20 UC), which means the NdNiO_3_ in‐plane lattice is strongly strained by that of the substrate lattice and approaches the bulk value gradually as the film thickness *t* increases. At *t* = 100 UC, *α* reaches the bulk values (the lattice mismatches between NdNiO_3_ bulk and the three substrates) (Figure 1b). Therefore, we can effectively tune the epitaxial strain in NdNiO_3_ films by choosing different substrates and controlling film thickness.

### 2.2 Polar Metallicity in NdNiO_3_(111)/LaAlO_3_(111) Films

**Figure 2:**
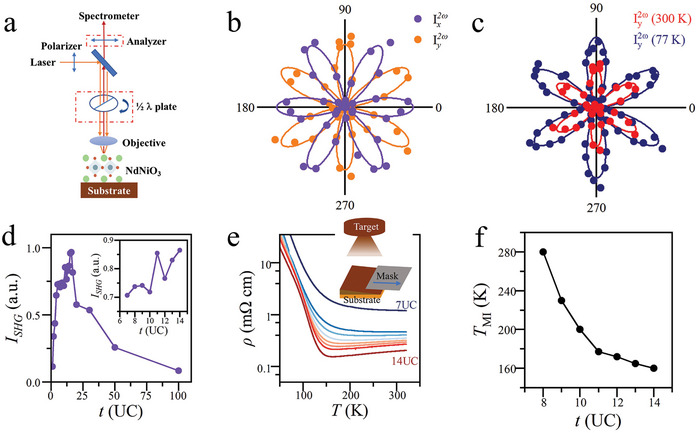
SHG polarimetry and electric transport measurements of NdNiO_3_(111) /LaAlO_3_(111). a) A schematic drawing of the SHG apparatus. The orange arrows represent the original light, and the red arrows are the second harmonic light. b) The SHG intensity $I_{x}^{2\omega }$ and $\;I_{y}^{2\omega }$ corresponds to the configurations that the analyzer's polarization are parallel or perpendicular to that of the polarizer, respectively, taken at *T* = 300 K. $I_{2\omega }^{x}\left(\phi \right)$ and $I_{2\omega }^{y}\left(\phi \right)$ resemble clear sixfold symmetry, where ϕ is the angle between the polarization of the incident light and NNO crystallographic $\left[1\bar{1}0\right]$ direction. ϕ varies continuously by rotating the *λ*/2 wave plate. c) $I_{2\omega }^{y}\left(\phi \right)$ at two representative temperatures, 300 and 77 K. For (c,d), the solid circles are experimental data and the solid curves are best fittings using *m* polar point group. d) The SHG intensity *I*_SHG_ as a function of NdNiO_3_ film thickness *t* taken from a NdNiO_3_/LaAlO_3_(111) wedge film. The inset is an amplified view of *I*_SHG_ for small *t*. e) The temperature‐dependent resistivity *ρ*(*T*) taken from the same wedge film. The inset is a schematic drawing of the synthesis of thickness‐wedge film using a mobile mask during PLD growth. f) The critical temperature *T*_MI_ of MIT retrieved from *ρ*(*T*) in e.

The electric polarization of NdNiO_3_/LaAlO_3_(111) films is measured by SHG apparatus (**Figure** **2**a). SHG method is sensitive to the breaking of inversion symmetry and is widely used to determine the point group symmetry of film or bulk crystal.^[^ ^21^ ^]^ In our setup, the incident light, which is linearly polarized after passing a polarizer, is normal to the film surface and its polarization is rotated in‐plane continuously by rotating a *λ*/2 (half‐wavelength) wave plate. The generated second harmonic signal passes the same *λ*/2 wave plate before it reaches the second polarizer‐the analyzer. The recorded SHG intensity $I_{2\omega }^{x}$ and $I_{2\omega }^{y}$ corresponds to the configurations with the polarization of the analyzer being parallel or perpendicular to that of the incident light. The measured angle‐dependent $I_{2\omega }^{x}\left(\phi \right)$ and $I_{2\omega }^{y}\left(\phi \right)$ show angular oscillations with 60° period in the polar coordinate system (Figure 2b). Here, ϕ is the angle between the polarization of the incident light and the NdNiO_3_ crystallographic $\left[1\bar{1}0\right]$ direction. $I_{2\omega }^{x}\left(\phi \right)$ and $I_{2\omega }^{y}\left(\phi \right)$ can be simultaneously fitted with the same parameters to the *m* point group. The detailed derivation can be found in the Experimental Section. Lowering the temperature to 77 K, the lowest temperature for our instrument, $I_{2\omega }^{x}\left(\phi \right)$ and $I_{2\omega }^{y}\left(\phi \right),$ increases in its amplitude while maintaining its symmetry (Figure 2c). Thus, the SHG signal and related electric polarization are robust from low temperatures to room temperature.

To systematically study the thickness dependence of the SHG signal and electric transport properties, we exploit the “thickness‐wedge” tactics on the basis of regular PLD technique. The “thickness‐wedge” growth of NdNiO_3_ films is realized by positioning a mask between the plume from the target and the substrate (inset of Figure 2e). The distance from the substrate to the mask is ≈1 mm to reduce the shadow effect. During film deposition, the mask is moving at a steady speed to generate a continuous variation of film thickness from one edge of the substrate to the other edge. As the change of the film thickness is in nm scale; while the length of the wedge is in mm scale, so the slope of the thickness wedge, which is determined by the deposition rate and the speed of the mask, is actually negligibly small. Thus, this tiny slope doesn't affect optical measurement performed on post‐growth films. By covering a whole range of film thickness on a single substrate, we not only greatly improve the throughput and efficiency but also significantly reduce the sample‐to‐sample variations due to inevitable variations in substrate precondition and growth condition.^[^ ^22^, ^23^ ^]^

The nonlinear optical measurements on the NdNiO_3_ wedge reveal the thickness dependence of SHG signals, which otherwise would be quite difficult to retrieve with the normal growth strategy. As the intensity of SHG signals depends on many factors, for example, the optical setups, notable discrepancy arises after we switch from one film to another and adjust the optical components. The SHG measurements on the wedge film, however, avoid this problem and we only need to move the sample to shine light on different locations for different thicknesses while keeping the optical components unchanged.

As the coherence length of SHG signal is much larger than our film thickness, the SHG signal from different depth simply adds up without phase interference. Thus, we can use the SHG signal *I*_SHG_, which is the amplitude of $I_{2\omega }^{x}\left(\phi \right)$ angular oscillations, to represent the electric polarization.^[^ ^24^, ^25^ ^]^ The thickness‐dependent *I*_SHG_(*t*), retrieved from a NdNiO_3_ wedge (Figure 2d), decreases fast with *t* and becomes relatively small for *t* > 50 UC, collaborating with the fact that the epitaxial strain on NdNiO_3_ film is relaxed for *t* > 50 UC (Figure 1d). It should be pointed out that the NdNiO_3_/LaAlO_3_ interface or the NdNiO_3_(111) surface lacks inversion symmetry so they generate SHG signals as well; this presumably is responsible for the small non‐zero *I*_SHG_ for the thick films. Concomitantly, the majority of SHG signals come from the NdNiO_3_ film itself. According to the previous electron density mapping studies of NdNiO_3_/LaAlO_3_(111),^[^ ^11^ ^]^ the relative polar displacement between Nd and O atoms is the source producing these SHG signals. Combining these facts together, one can conclude that the SHG signal is a direct measurement of the electric polarization in the NdNiO_3_ films.

The resistivity of NdNiO_3_ films *ρ*(*T*) shows characteristic MIT (Figure 2e), consistent with reports that in RNiO_3_ (R: La, Pr, Nd, Sm, etc.), the high temperature paramagnetic metallic state transits into low temperature antiferromagnetic insulating state through first‐order phase transition.^[^ ^9^, ^23^, ^26^, ^27^, ^28^, ^29^, ^30^ ^]^ Apparently, electric polarization coexists with both the metallic and insulating states. Moreover, the critical temperature *T*_MI_ of MIT depends on the film thickness *t* and shifts significantly to lower temperature as *t* increases (Figure 2f). This is accompanied by a substantial drop in *ρ*, for example, *ρ* of the metallic state reduces by an order of magnitude as *t* increases from 7 UC to 14 UC. Thus, metallicity is enhanced in thicker films. Ultrathin LaNiO_3_ film is known to transit into an insulating state that is ascribed to emergence of a static charge/spin order as the dimensionality of LaNiO_3_ lowers from 3D to 2D.^[^ ^30^ ^]^ This is consistent with the strengthening of insulating behavior at NdNiO_3_ thinner films. Variations of film strain, and consequently, the distortion of lattice structure may contribute to *T*_MI_(*t*) dependence as well.

### 2.3 Strain Effect on Polar Metallicity in NdNiO_3_(111) Films on Different Substrates

**Figure 3:**
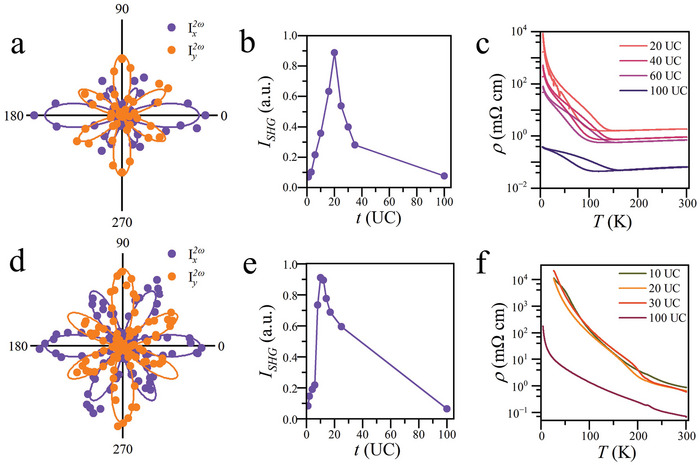
Induced electric polarization in NdNiO_3_(111)/YAlO_3_(101) and NdNiO_3_(111)/NdGaO_3_(101). a) $I_{2\omega }^{x}\left(\phi \right)$ and $I_{2\omega }^{y}\left(\phi \right)$ of NdNiO_3_(20 UC)/YAlO_3_(101) with compressive epitaxial strain at *T* = 300 K. The solid curves are the best fittings of experimental data (solid circles) based on *m* polar point group symmetry. b,c) The SHG intensity *I*_SHG_ and *ρ*(*T*) as a function of NdNiO_3_ thickness of NdNiO_3_/YAlO_3_(101). d–f) The same as (a–c), except for NdNiO_3_/NdGaO_3_(101) with tensile epitaxial strain. In (f), NdNiO_3_ is insulating till room temperature.

To investigate whether larger epitaxial strain can further enhance the effect, we carry out SHG polarimetry and electric transport measurements on NdNiO_3_/YAlO_3_(101) (**Figure** **3**a–c) for the lattice mismatch, and the resultant compressive strain on NdNiO_3_ film is much bigger in NdNiO_3_/YAlO_3_(101) than in NdNiO_3_/LaAlO_3_(111) (Figure 1d). The SHG signals $I_{2\omega }^{x}\left(\phi \right)$ and $I_{2\omega }^{y}\left(\phi \right)$ have angular oscillations with 60^°^ period but the oscillation amplitudes no longer have sixfold symmetry like NdNiO_3_/LaAlO_3_(111) though they can still be well fitted to *m* point group. The difference is due to the fact that the lattice of YAlO_3_(101) surface, and consequently, the NdNiO_3_ film grown on top, is slightly elongated along $\left[1\bar{1}0\right]$ direction and loses sixfold rotational symmetry. The thickness dependent *I*_SHG_(*t*) shows that the strain effect is maximized at about 20 UC (Figure 3b), similar to NdNiO_3_/LaAlO_3_(111). The MIT in *ρ*(*T*) persists in NdNiO_3_/YAlO_3_(101) and the polar metallicity is present for *T* > *T*_MI_ (≈150 K) (Figure 3c).

Then, we alter the epitaxial strain from compressive to tensile by switching to NdGaO_3_(101) substrate (Figure 1a). $I_{2\omega }^{x}\left(\phi \right)$ and $I_{2\omega }^{y}\left(\phi \right)$ of NdNiO_3_/NdGaO_3_(101) are similar to that of NdNiO_3_/YAlO_3_(101) and follow the symmetry of *m* point group (Figure 3d). *I*_SHG_(*t*) peaks at thinner film (≈10 UC), indicating the compressive strain can also induce electric polarization (Figure 3e). The resistivity *ρ*(*T*), however, has a negative slope for all temperatures (Figure 3f), implying the NdNiO_3_ film is insulating. We have also synthesized single crystalline pesudocubic NdNiO_3_(111) film on SrTiO_3_(111) (STO) substrate, which provides tensile strain but with larger lattice mismatch (≈2.4%) compared to NdGaO_3_(101). NdNiO_3_/SrTiO_3_(111) produces no observable SHG signal and is insulating from room temperature down to 3 K (see the Supporting Information for details). Thus, the tensile strain is not effective in inducing NdNiO_3_ polar metal state.

**Figure 4:**
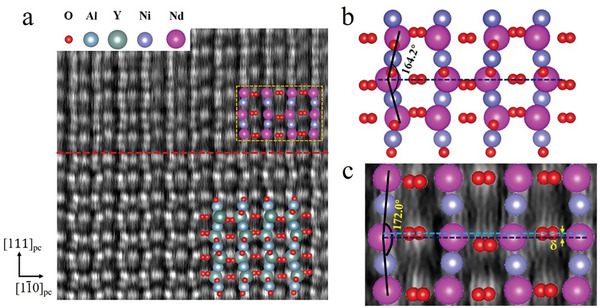
HAADF‐STEM image taken on NdNiO_3_(111)/YAlO_3_(101). a) STEM image shows the atomic locations of all species of atoms. The red dashed line represents the interface between NdNiO_3_ and YAlO_3_. b) A schematic drawing of lattice structure of NdNiO_3_(11$\bar{2}$)_pc_ plane in its bulk form is shown for comparison. c) A magnified view of the area in the red box in panel (a). The navy and blue dashed lines correspond to the lines passing through the center of Nd atom and O atom, respectively. *δ* ≈ 0.23Å is the displacement of Nd atom with respect to O atom. The Nd─Nd angle in NNO film is increased from 164.2° (bulk) to 172.0° due to the interfacial strain.

It is experimentally challenging to obtain the atomic image of oxygen atoms by scanning transmission electron microscopy (STEM), and NdNiO_3_ samples degrade over time when exposed to high energy electron beam. With effort and luck, we succeed in getting atomic‐resolved STEM images on NdNiO_3_/YAlO_3_(101). The polar displacement between Nd and O atoms is ≈0.23Å (**Figure** **4**), giving rise to the SHG signals in Figure 3a. Meanwhile, the zigzag arrangement of the Nd─Nd─Nd chain along the [111] direction is constrained by the interfacial epitaxial compressive strain, increasing the Nd─Nd angles toward a straight line. This, in turn, induces polar displacements.

### 2.4 An Ideal Polar Metal State Induced by Anisotropic Strain in NdNiO_3_(102)/ YAlO_3_(111) Films

**Figure 5:**
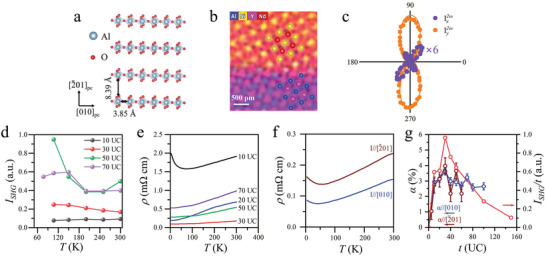
Suppressed MIT and induced electric polarization in NdNiO_3_(102)/YAlO_3_(111) under anisotropic compressive strain. a) A schematic drawing of YAlO_3_(111) plane shows its lattice is in rectangle shape. b) Atomic resolution chemical mapping by energy dispersive X‐ray spectroscopy (EDS) of STEM on NdNiO_3_/YAlO_3_(111). c) $I_{2\omega }^{x}\left(\phi \right)$ and $I_{2\omega }^{y}\left(\phi \right)$ (multiplied by six times for visibility) of NdNiO_3_(20 UC)/YAlO_3_(111) at *T* = 300 K. The solid curves are the best fittings of experimental data (solid circles) based on *m* polar point group symmetry. d) *I*_SHG_ as a function of temperature *T* for several representative NdNiO_3_ thicknesses. e) *ρ*(*T*) along NdNiO_3_ pesudocubic [010] direction evidences that MIT is completely suppressed and the NdNiO_3_ films are metallic even at low temperatures for *t* ≥ 20 UC. f) *ρ*(*T*) for 30 UC NdNiO_3_ film is anisotropic along [010] and $\left[\bar{2}01\right]$ directions, in response to the anisotropic in‐plane strain. g) The lattice mismatch *α*(*t*), along both the [010] and $\left[\bar{2}01\right]$ directions, and the thickness‐normalized SHG intensity *I*_SHG_ */t*(*t*) show clear correlation in *t*‐dependence, indicating the electric polarization is induced by epitaxial strain.

So far, the epitaxial strain from LaAlO_3_(111), YAlO_3_(101), and NdGaO_3_(101) substrates is along pseudocubic NdNiO_3_(111) plane, and we can generalize this idea to test whether strain along other planes may have similar effects. By using YAlO_3_(111) substrate as an example, we have grown pseudocubic NdNiO_3_(102) film on top. The in‐plane lattice of YAlO_3_(111) and NdNiO_3_(102) forms unit cells with rectangle shape (**Figure** **5**a), and the STEM image shows high‐quality epitaxy growth of NdNiO_3_/YAlO_3_(111) at atomic scale (Figure 5b). It is a pity that all species of atoms are resolved in STEM images except oxygen atoms that prevent us from measuring the atomic displacement directly (see the Supporting Information for details). However, substantial SHG signal is generated by NdNiO_3_/YAlO_3_(111). $I_{2\omega }^{x}\left(\phi \right)$ and $I_{2\omega }^{y}\left(\phi \right)$ of NdNiO_3_(102)_pc_ cannot be fitted to *m* point group but have to be fitted with *1* point group (Figure 5c), indicating lower symmetry due to anisotropic epitaxial strain. The SHG signal is robust against thermal fluctuations and survives till 100 UC, evidenced by the temperature‐ and thickness‐dependent *I*_SHG_ (Figure 5d). More importantly, MIT is suppressed for thicker film (*t* ≥ 20 UC) and the metallic behavior of *ρ*(*T*), which is measured along NdNiO_3_ pesudocubic $\left[1\bar{1}0\right]$ direction, remains till 3 K, the lowest temperature we can reach (Figure 5e). Thus, this is a true polar metal ground state. Remarkably, for the most metallic 30 UC NdNiO_3_, the resistivity at room temperature *ρ*(*T = 300 K*) ≈ 173 µΩ cm, and this is low enough to be comparable to some metals. Meanwhile, the appreciable anisotropy in *ρ*(*T*) along the [010] and $\left[\bar{2}01\right]$ directions (Figure 5f) collaborates with the anisotropic in‐plane strain imposed by the interfacial rectangle lattice of the YAlO_3_(111) substrate. Thus, NdNiO_3_/YAlO_3_(111) is a good candidate for polar metal applications.

To shine light on the close connection between the epitaxial strain and the induced electric polarization, we made a direct comparison between the thickness‐dependent lattice mismatch *α*(*t*) and *I*_SHG_/*t*. Here *I*_SHG_/*t* is used as an indicator of SHG signals generated from every unit cell of NdNiO_3_, which is proportional to the electric polarization. Apparently, *α*(*t*) along [010] and $\left[\bar{2}01\right]$ directions are correlated with *I*_SHG_/*t* (Figure 5g). Therefore, the peaks in *I*_SHG_(*t*) are due to the variations of epitaxial strains built in NdNiO_3_ films.

From the RHEED, X‐ray reflectivity, rocking curve, and AFM measurements, we can conclude that the crystalline quality and morphology of NdNiO_3_(102) film are similar to NdNiO_3_(111) films. In addition, the epitaxy growth of NdNiO_3_(102) film is performed in the same PLD chamber with the same growth recipe (except that the preconditioning of the substrates is different). It is highly unlikely that these extrinsic factors are the reasons for better metallicity in NdNiO_3_(102) film. Instead, the suppression of MIT is the key to the metallic ground state at all temperatures. Previous studies show that MIT in NdNiO_3_ bulk is associated with a first‐order structural phase transition.^[^ ^31^, ^32^ ^]^ As the temperature goes below *T*_MI_, the volume of NdNiO_3_ unit cell expands about 0.2%, which results from a small increase in the Ni─O distance (≈0.2%) and a simultaneous decrease of the Ni─O─Ni bond angle. For NdNiO_3_(111) films on LaAlO_3_(111) and YAlO_3_(101), the compressive epitaxial strain straightens the Ni─O─Ni bond, and thus lowers *T*_MI_. In contrast, the tensile strain increases *T*_MI_ in NdNiO_3_(111)/NdGaO_3_(101). When the compressive strain becomes anisotropic in‐plane in NdNiO_3_(102)/YAlO_3_(111), it lowers the NdNiO_3_ lattice symmetry further from polar *m* group to polar *1* point group. We postulate that the lower symmetry and constraints imposed by bonding across NdNiO_3_ and YAlO_3_ interfaces make it geometrically more difficult to expand all the Ni─O bonds and tilt all Ni─O octahedral, simultaneously. As a result, the structural phase transition is suppressed and MIT diminishes in NdNiO_3_(102) /YAlO_3_(111), giving rise to a polar metal state at all temperatures.

It should also be mentioned that we observe no contrast for polar domains under the SHG microcopy for all the NdNiO_3_ films we've studied. This is consistent with no domain contrast under piezoresponse force microscopy (PFM). As an attempt to flip the polarization direction, a dc voltage is applied between the PFM tip and the film during tip scanning. As the tip voltage is ramped from −20 to 20 V, no sign of flip is observed. This, however, is no surprise. The absence of polar domains under PFM in fact is also quite common among the reported polar metallic materials, for example, LiOsO_3_ and BaTiO_3_/SrTiO_3_/LaTiO_3_ superlattices.^[^ ^6^, ^12^ ^]^ For metallic NdNiO_3_ films, the electric field applied by the tip is screened inside the films. This group of polar materials, which lacks switchability, is given the name “polar metal” in the literature (to be distinguished from “ferroelectric metal”), and our NdNiO_3_ films fall into this category.

## 3 Conclusion 

In conclusion, electric polarization of NdNiO_3_ can be induced by epitaxial strain on the LaAlO_3_(111), YAlO_3_(101), NdGaO_3_(101), and YAlO_3_(111) substrates. The metallicity and MIT remain in NdNiO_3_(111)/LaAlO_3_(111) and NdNiO_3_(111)/YAlO_3_(101). Remarkably, the metallicity is enhanced in NdNiO_3_(102)/YAlO_3_(111) and becomes the ground state even at low temperatures. The electric polarization can be further engineered by controlling the NdNiO_3_ film thickness and consequently the epitaxial strain. We conclude that the idea of induction of electric polarization by epitaxial strain is rather general and applicable to many perovskite oxides no matter if the strain is compressive or tensile, isotropic or anisotropic. These together open the route to design and synthesize multi‐functional materials by combining induced electric polarization with other attractive properties, for example, ferro‐ or antiferro‐magnetism and superconductivity.

## 4 Experimental Section

### Film Growth

Perovskite oxide NdNiO_3_ was deposited on LaAlO_3_, NdGaO_3_, and YAlO_3_ substrates by PLD. A KrF excimer laser with 248 nm wavelength was used, with a repetition rate of 2 Hz. The substrates were heated up to 640 °C and oxygen partial pressure was kept at 100 mTorr during growth. The laser fluence was 1.44 J cm^−2^. A mobile mask was inserted between the target and the substrate. The slope of the film thickness wedge was controlled by the deposition rate and the speed of the mask.

### Structural Characterization

The film deposition was monitored by in situ RHEED apparatus. To characterize the lattice structure of both NdNiO_3_ films and substrates, high‐resolution X‐ray diffraction instrument (HR‐XRD, Bruker, D8 discover) was used with monochromatic Cu Kα1 radiation (*λ* = 1.5406 Å).

### Electronic Transport Measurement

NdNiO_3_ films were patterned by standard UV‐lithography and ion milling to form Hall bar devices with 300 µm length and 100 µm width. Gold contact pads were attached to Hall bars for good Ohmic contact. High‐throughput four‐point measurements were carried out by using Keithley 2450 and Keithley DAQ 6510 electronics. All measurements were performed in a closed‐cycle cryocooler to reach temperatures as low as 3 K.

### Non‐Linear Optics

SHG signals were generated in a confocal microscope (WITec, Alpha300RAS) with 1064 nm laser excitation (NPI Rainbow1064 OEM). As shown in Figure 2a, the polarization of the incident light was rotated continuously in‐plane and it made the angle ϕ with respect to the NdNiO_3_ pesudocubic $\left[1\bar{1}0\right]$ direction, so the corresponding electric field was given by

(1) $$\left[\begin{array}{c} E_{x}\\ E_{y}\;\\ E_{z} \end{array}\right]=\left[\begin{array}{c} E_{0}\cos\phi \\ E_{0}\sin\phi \\ 0 \end{array}\right]\;$$

The SHG *d* matrix for the *m* point group with electric polarization in ($1\bar{1}0$) plane was

(2) $$d_{m}=\left(\begin{array}{cccccc} 0 & 0 & 0 & 0 & d_{15} & d_{16}\\ d_{21} & d_{22} & d_{23} & d_{24} & 0 & 0\\ d_{31} & d_{32} & d_{33} & d_{34} & 0 & 0 \end{array}\right)\;$$

So the SHG signal became

(3) $$P^{2\omega }=d_{m}\;\left(\begin{array}{c} E_{0}^{2}\cos^{2}\phi \\ E_{0}^{2}\sin^{2}\phi \\ 0\\ 0\\ 0\\ 2E_{0}^{2}\cos\phi \sin\phi \end{array}\right)=E_{0}^{2}\;\left(\begin{array}{c} -2d_{16}\sin\phi \cos\phi \\ d_{21}\mathrm{co}s^{2}\phi +d_{22}\mathrm{si}n^{2}\phi \\ d_{31}\mathrm{co}s^{2}\phi +d_{32}\mathrm{si}n^{2}\phi \end{array}\right)$$

As the SHG light went through a *λ*/2 wave plate before it reached the analyzer, the SHG light intensities corresponding to *x*‐ and *y*‐polarization of the analyzer were:

(4) $$I_{x}^{2\omega }\propto E_{0}^{4}{\left(d_{21}\sin\phi \cos^{2}\phi +d_{22}\mathrm{si}n^{3}\phi -2d_{16}\sin\phi \cos^{2}\phi \right)}^{2}$$

(5) $$I_{y}^{2\omega }\propto E_{0}^{4}{\left(d_{21}\mathrm{co}s^{3}\phi +d_{22}\mathrm{si}n^{2}\phi \cos\phi +2d_{16}\mathrm{si}n^{2}\phi \cos\phi \right)}^{2}$$

The above expression fits well with the SHG data taken from NdNiO_3_(111)/LaAlO_3_(111), NdNiO_3_(111)/YAlO_3_(101), and NdNiO_3_(111)/NdGaO_3_(101).

For NdNiO_3_(102)_pc_/YAlO_3_(111), the SHG *d* matrix for the *1* point group had to be used:

(6) $$d_{1}=\left(\begin{array}{cccccc} d_{11} & d_{12} & d_{13} & d_{14} & d_{15} & d_{16}\\ d_{21} & d_{22} & d_{23} & d_{24} & d_{25} & d_{26}\\ d_{31} & d_{32} & d_{33} & d_{34} & d_{35} & d_{36} \end{array}\right)\;$$

Then, the SHG signal became

(7) $$P^{2\omega }=d_{1}\;\left(\begin{array}{c} E_{0}^{2}\cos^{2}\phi \\ E_{0}^{2}\sin^{2}\phi \\ 0\\ 0\\ 0\\ 2E_{0}^{2}\cos\phi \sin\phi \end{array}\right)=E_{0}^{2}\;\left(\begin{array}{c} d_{11}\cos^{2}\phi +d_{12}\sin^{2}\phi -2d_{16}\sin\phi \cos\phi \\ d_{21}\cos^{2}\phi +d_{22}\sin^{2}\phi -2d_{26}\sin\phi \cos\phi \\ d_{31}\cos^{2}\phi +d_{32}\sin^{2}\phi -2d_{36}\sin\phi \cos\phi \end{array}\right)$$

So

(8) $$\begin{array}{ccc} I_{x}^{2\omega } & \propto & E_{0}^{4}d_{11}\mathrm{co}s^{3}\phi +d_{12}\mathrm{si}n^{2}\phi \cos\phi -2d_{16}\sin\phi \mathrm{co}s^{2}\phi +d_{21}\sin\phi \mathrm{co}s^{2}\phi \\ & & +{d_{22}\mathrm{si}n^{3}\phi -2d_{26}\mathrm{si}n^{2}\phi \cos\phi }^{2} \end{array}$$

(9) $$\begin{array}{ccc} I_{y}^{2\omega } & \propto & E_{0}^{4}(-d_{11}\sin\phi \mathrm{co}s^{2}\phi -d_{12}\mathrm{si}n^{3}\phi +2d_{16}\mathrm{si}n^{2}\phi \cos\phi +d_{21}\mathrm{co}s^{3}\phi \\ & & +d_{22}\mathrm{si}n^{2}\phi \cos\phi -2d_{26}\sin\phi \mathrm{co}s^{2}{\phi )}^{2} \end{array}$$

## Conflict of interest

The authors declare no conflict of interest.

## Supporting information

Supporting Information

## Data Availability

The data that support the findings of this study are available from the corresponding author upon reasonable request.
