# Supplementary material for: Polar Metallicity Controlled by Epitaxial Strain Engineering
Source: Adv Sci (Weinh). 2024 Aug 29;11(40):2408329. doi: 10.1002/advs.202408329 (PMC11516258; doi:10.1002/advs.202408329)
Supplement: Supplementary file 1 — Supporting Information [file ADVS-11-2408329-s001.docx]

**Polar metallicity controlled by epitaxial strain engineering**

Mingdong Dong^1,2,3,4^, Yichi Zhang^1,2,3,4^, Jing-ming Cao^5^, Haowen Chen^6^, Qiyang Lu^6^, Hong-fei Wang^5^, Jie Wu^1,2,3^ *

*^1^Department of Physics, School of Science, Westlake University, Hangzhou 310030, China*

*^2^Research Center for Industries of the Future, Westlake University, Hangzhou31 0030, China*

*^3^Key Laboratory for Quantum Materials of Zhejiang Province, School of Science, Westlake University, Hangzhou, 310030, China*

*^4^School of Physics, Zhejiang University, Hangzhou 310027, China*

*^5^Department of Chemistry, School of Science, Westlake University, Hangzhou 310030, China*

*^6^School of Engineering, Westlake University, Hangzhou 310030, China*

**Author to whom correspondence should be addressed:* [*wujie@westlake.edu.cn*](mailto:wujie@westlake.edu.cn)

**Supplementary information**

**1. Scanning tunneling electron microscope (STEM) images taken from NdNiO_3_(102)_pc_/YAlO_3_(111)**

It is experimentally challenging to obtain the atomic image of oxygen atoms by STEM and NdNiO_3_ samples degrade over time when exposed to high energy electron beam. For NdNiO_3_(102)_pc_/YAlO_3_(111), we managed to obtain high resolution images (Fig. S1) but we couldn’t identify the locations of oxygen atoms in the NNO film despite of the efforts we devoted. The Nd-Nd chain along the [102]_pc_ direction is straightened due to the epitaxial strain and this, in turn, induces electric polarization.


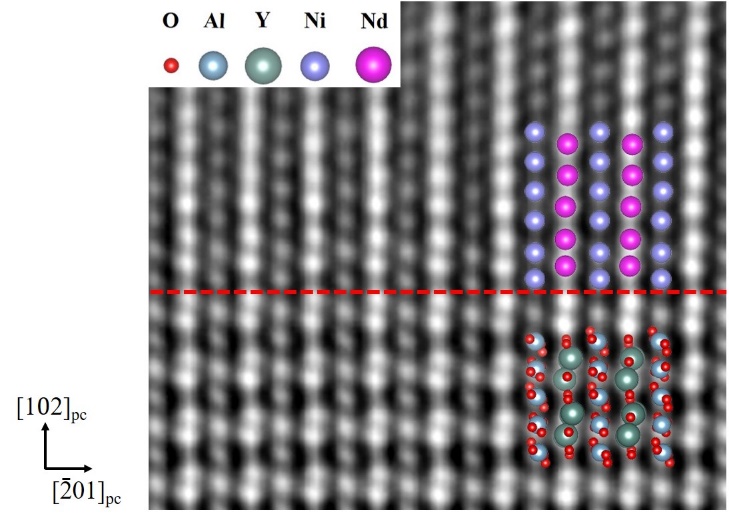


**Figure S1 |** **iDPC-STEM image taken on NdNiO_3_(102)_pc_/YAlO_3_(111)**. An iDPC-STEM images captured along the [010]_pc_ zone axis in cross-sectional view. The red dashed line represents the interface between NdNiO_3_ and YAlO_3_. The interfacial epitaxial compressive strain causes the Nd atoms to align in a straight line in the [102]_pc_ direction.

**2. Structural characterization of NdNiO_3_ films by X-ray**

The rocking curves of X-ray diffraction (XRD) are shown in Fig. S2 and the X-ray reflectivity (XRR) curves in Fig. S3. Apparently, the FWHM values of the XRD peaks are all close to 0.04°, which reflects high quality crystalline structure and good epitaxy growth. The intensity of the XRR curves oscillates with the incident angle due to the interference between the film surface and interface. The film thickness, retrieved from the oscillation period, agrees nicely with the thickness calibrated during film deposition (except NdNiO_3_/NdGaO_3_(101), whose oscillation is nearly invisible). These results evidence the high film quality for epitaxy on different substrates.


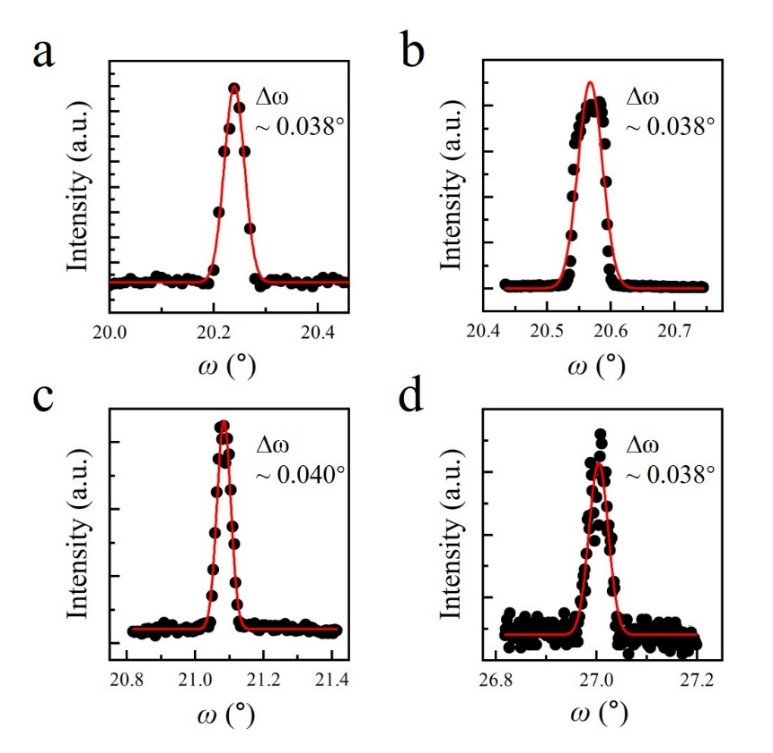


**Figure S2 |** **Rocking curves taken from (a) 60 UC NdNiO_3_/YAlO_3_(101), (b) 100 UC NdNiO_3_/LaAlO_3_(111), (c) 100 UC NdNiO_3_/NdGaO_3_(101), and (d) 70 UC NdNiO_3_ /YAlO_3_(111).** Solid circles are experimental data and the red curve is the best fitting based on Gaussian distribution. The corresponding FWHM value *Δω* of every XRD peak is also shown.


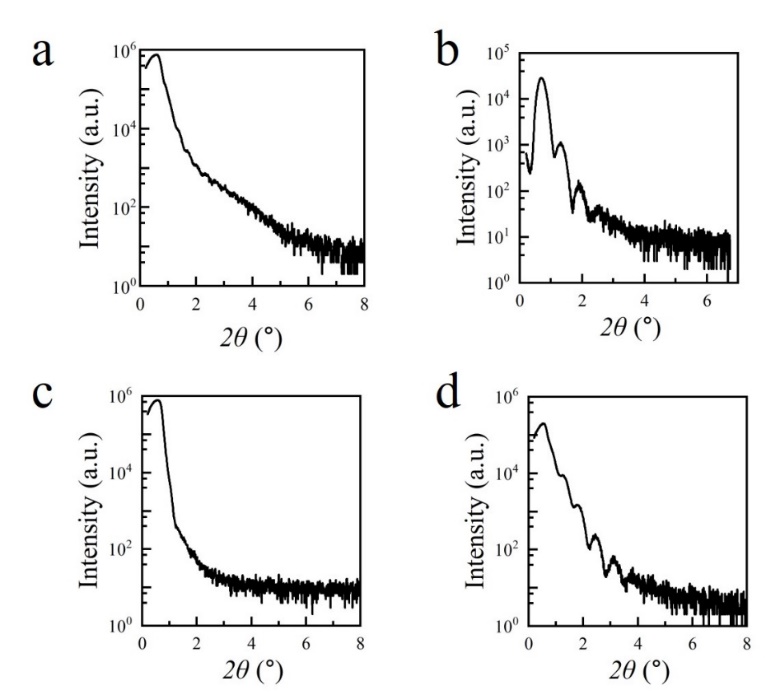


**Figure S3 |** **XRR curves taken from (a) 60 UC NdNiO_3_/YAlO_3_(101), (b) 100 UC NdNiO_3_/LaAlO_3_(111), (c) 100 UC NdNiO_3_/NdGaO_3_(101), and (d) 70 UC NdNiO_3_ /YAlO_3_(111).**

**3. Morphology of NdNiO_3_ films by atomic force microscopy (AFM)**

We performed AFM to characterize the surface roughness of the NdNiO_3_ films. Atomic steps are clearly visible for NdNiO_3_ films in Fig. S4, implying a nice layer-by-layer growth mode. The RMS of the NdNiO_3_ films on YAlO_3_(101), LaAlO_3_(111), NdGaO_3_(101) and YAlO_3_(111) substrates are 0.18 nm, 0.7 nm, 0.8 nm and 0.2 nm, respectively. These values are close to the *c*-axis constant of NdNiO_3_ film (~ 0.22 nm for NdNiO_3_(111) and 0.17 nm for NdNiO_3_(102)), implying that NdNiO_3_ films are atomically flat.

In addition, we studied the effect of atomic steps on polar metal state by deliberately choosing two substrates with quite different density of atomic steps due to the miscut of substrates (Figs. S4d and S4e). The NdNiO_3_ films deposited on these two substrates, however, show similar behavior in terms of electric transport and non-linear optical measurements (Figs. S4f and S4g). This illustrates that the polar metal state is not affected by the presence of steps, and it is intrinsic to NdNiO_3_ films.

*
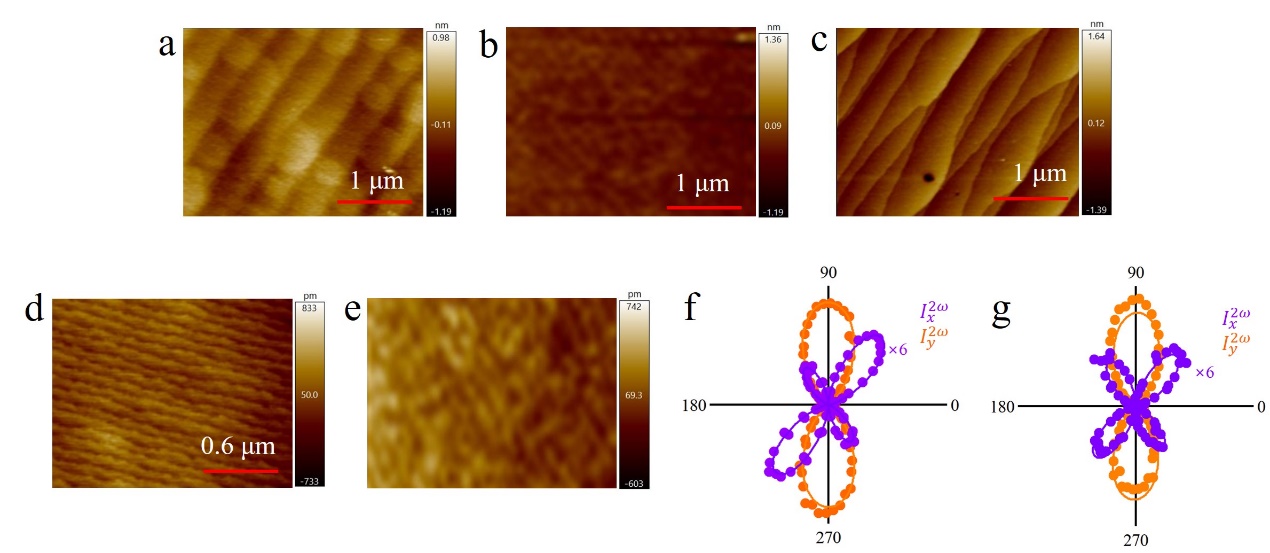
*

**Figure S4 | Atomic force microscopy (AFM) images on (a) NdNiO_3_(15 UC)/YAlO_3_(101), (b) NdNiO_3_(15 UC)/LaAlO_3_(111), and (c) NdNiO_3_(15 UC)/NdGaO_3_(101), (d), and (e), two NdNiO_3_(30 UC)/YAlO_3_(111) with different density of atomic steps.** **f**, and **g**, The second harmonic generation (SHG) signals from these two NdNiO_3_(30 UC)/YAlO_3_(111) samples, however, show very similar behavior. This evidences that the electric polarization is insensitive to atomic steps and is intrinsic to NdNiO_3_ films.

**4. Absence of polar domain on NdNiO_3_ films by piezoresponse force microscopy (PFM)**

We tried PFM and observed no contrast for polar domains (Fig.S5). We also tried to flip the polarization direction by applying a dc voltage between the PFM tip and the film. As the tip voltage was ramped from -20 to 20V, we did not observe the flip. Since the NNO film becomes metallic, the electric field applied by the tip is screened inside the film.

*
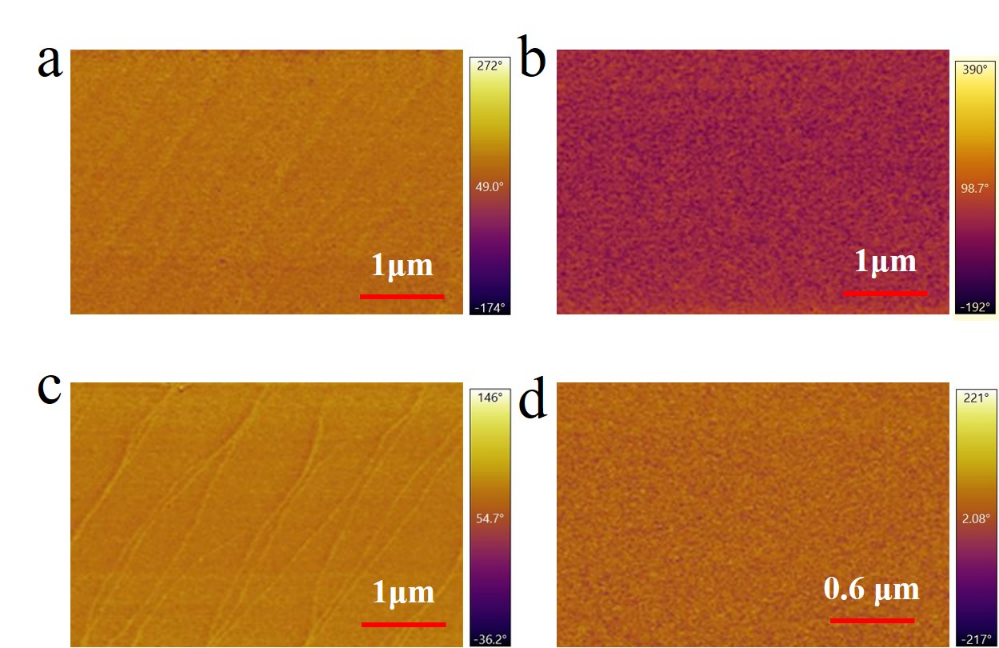
*

**Figure S5 | PFM phase images on (a) NdNiO_3_(15 UC)/YAlO_3_(101), (b) NdNiO_3_(15 UC)/LaAlO_3_(111), (c) NdNiO_3_(15 UC)/NdGaO_3_(101), and (d) NdNiO_3_(30 UC)/YAlO_3_(111).**

**5. Structural, electronic and non-linear optical properties of NdNiO_3_(100 UC)/SrTiO_3_(111)**

For NdNiO_3_/SrTiO_3_(111), its lattice structure, electric transport and SHG measurements are shown in Fig. S6. XRD illustrates the epitaxial nature of NdNiO_3_(111)_pc_ on SrTiO_3_(111). It is clear that NdNiO_3_/SrTiO_3_(111) is insulating and shows no noticeable SHG signal so NdNiO_3_/SrTiO_3_(111) is non-polar under tensile strain when deposited onto SrTiO_3_(111) substrate.


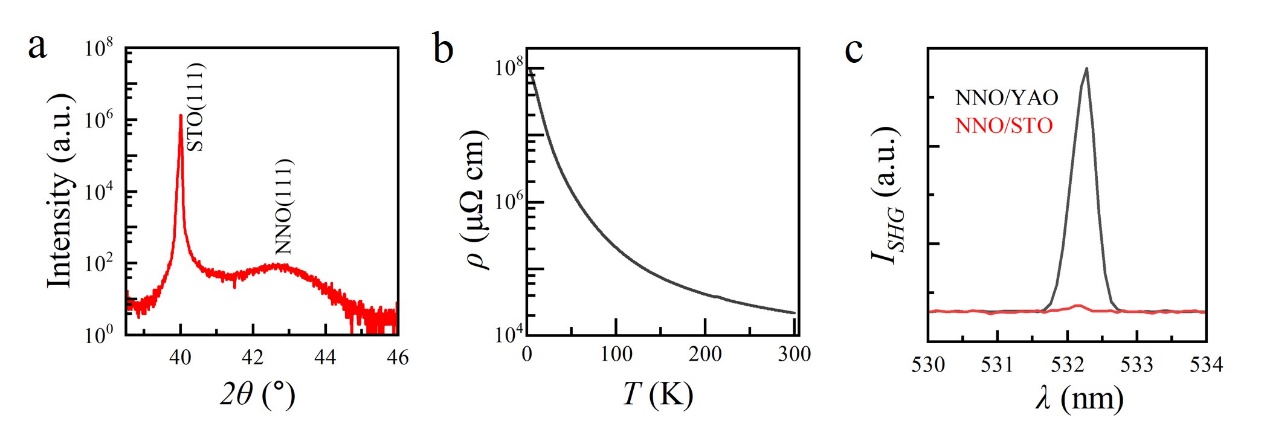


**Figure S6 | Structural, electronic and non-linear optical properties of NdNiO_3_(100 UC)/SrTiO_3_(111).** **a**, XRD spectrum shows NdNiO_3_(111)_pc_ is epitaxially grown on top of SrTiO_3_(111) substrate. **b**, The temperature-dependent resistivity *ρ*(*T*) is insulating from low temperatures to room temperature. **c**, Comparison of SHG signals from NdNiO_3_/YAlO_3_(111) and NdNiO_3_/SrTiO_3_(111) films clearly shows that the former is polar and the latter is non-polar. The laser wavelength is 1064.5 nm so the wavelength of the second harmonic signal is 532.25 nm, which is nearly absent from the NdNiO_3_/SrTiO_3_(111) film while it is substantial from the NdNiO_3_/YAlO_3_(111) film.
